# Clioquinol Decreases Levels of Phosphorylated, Truncated, and Oligomerized Tau Protein

**DOI:** 10.3390/ijms222112063

**Published:** 2021-11-08

**Authors:** Gaoping Lin, Feiyan Zhu, Nicholas M. Kanaan, Rei Asano, Norimichi Shirafuji, Hirohito Sasaki, Tomohisa Yamaguchi, Soichi Enomoto, Yoshinori Endo, Asako Ueno, Masamichi Ikawa, Kouji Hayashi, Osamu Yamamura, Shu-Hui Yen, Yasunari Nakamoto, Tadanori Hamano

**Affiliations:** 1 Second Department of Internal Medicine, Faculty of Medical Sciences, University of Fukui, Fukui 910-1193, Japan; lingaoping@hmc.edu.cn (G.L.); zhu@u-fukui.ac.jp (F.Z.); asanorei0718@gmail.com (R.A.); sira@u-fukui.ac.jp (N.S.); hsasaki@u-fukui.ac.jp (H.S.); tomohisa@u-fukui.ac.jp (T.Y.); weltraum@u-fukui.ac.jp (S.E.); yendo@u-fukui.ac.jp (Y.E.); maedaa@u-fukui.ac.jp (A.U.); iqw@u-fukui.ac.jp (M.I.); khayashi@fukui-hsu.ac.jp (K.H.); kapi@u-fukui.ac.jp (O.Y.); nakamoto-med2@med.u-fukui.ac.jp (Y.N.); 2 Department of Neurology, Zhejiang Provincial People’s Hospital, People’s Hospital of Hangzhou Medical College, Hangzhou 310014, China; 3 Department of Neurology, Tongde Hospital of Zhejiang Province, Hangzhou 310012, China; 4 Department of Translational Neuroscience, College of Human Medicine, Michigan State University, Grand Rapids, MI 49503, USA; nkanaan01@gmail.com; 5 Department of Aging and Dementia (DAD), Faculty of Medical Sciences, University of Fukui, Fukui 910-1193, Japan; 6 Department of Neuroscience, Mayo Clinic Jacksonville, Jacksonville, FL 32224, USA; shusam@icloud.com; 7 Life Science Innovation Center, University of Fukui, Fukui 910-1193, Japan

**Keywords:** Alzheimer’s disease, clioquinol, tau protein, tau oligomer, JNK, PP2A, p38MAPK, metal protein attenuating compounds, autophagy

## Abstract

The neuropathological hallmarks of Alzheimer’s disease (AD) are senile plaques (SPs), which are composed of amyloid β protein (Aβ), and neurofibrillary tangles (NFTs), which consist of highly phosphorylated tau protein. As bio-metal imbalance may be involved in the formation of NFT and SPs, metal regulation may be a direction for AD treatment. Clioquinol (CQ) is a metal-protein attenuating compound with mild chelating effects for Zn^2+^ and Cu^2+^, and CQ can not only detach metals from SPs, but also decrease amyloid aggregation in the brain. Previous studies suggested that Cu^2+^ induces the hyperphosphorylation of tau. However, the effects of CQ on tau were not fully explored. To examine the effects of CQ on tau metabolism, we used a human neuroblastoma cell line, M1C cells, which express wild-type tau protein (4R0N) via tetracycline-off (TetOff) induction. In a morphological study and ATP assay, up to 10 μM CQ had no effect on cell viability; however, 100 μM CQ had cytotoxic effects. CQ decreased accumulation of Cu^+^ in the M1C cells (39.4% of the control), and both total and phosphorylated tau protein. It also decreased the activity of c-Jun N-terminal kinase (JNK) and p38 mitogen-activated protein kinase (p38 MAPK) (37.3% and 60.7% levels of the control, respectively), which are tau kinases. Of note, activation of protein phosphatase 2A (PP2A), which is a tau phosphatase, was also observed after CQ treatment. Fractionation experiments demonstrated a reduction of oligomeric tau in the tris insoluble, sarkosyl soluble fraction by CQ treatment. CQ also decreased caspase-cleaved tau, which accelerated the aggregation of tau protein. CQ activated autophagy and proteasome pathways, which are considered important for the degradation of tau protein. Although further studies are needed to elucidate the mechanisms responsible for the effects of CQ on tau, CQ may shed light on possible AD therapeutics.

## 1. Introduction

The characteristic pathological hallmarks of Alzheimer’s disease (AD) are neurofibrillary tangles (NFTs) that consist of intracellular hyperphosphorylated tau [1], and senile plaques (SPs) formed by the aggregation of extracellular amyloid β peptide (Aβ) [2]. Currently, drugs for AD mainly consist of acetylcholine esterase inhibitors (donepezil, galantamine, and rivastigmine) [3] and N-methyl-D-aspartic acid receptor (NMDA) antagonists [4]. These drugs can alleviate the symptoms of AD, but do not reverse or stop the course of AD. Therefore, it is necessary to find drugs that can affect the pathological changes in the AD brain.

Bio-metal imbalance in the brain may be associated with pathological changes in AD. First, the concentrations of Zn^2+^ and Cu^2+^ in SPs are 9–10 times higher than in the surrounding tissues [5]. Second, Zn^2+^ and Cu^2+^ mediate Aβ aggregation and toxicity through the production of hydrogen peroxide and free radicals [6]. Tau protein co-deposits with several transition metals [7], and compromised metal homeostasis is closely linked with AD and tauopathy [2,8,9,10]. In addition, Cu^2+^ induces hyperphosphorylation of tau and promotes its aggregation [11]. Zinc can induce hyperphosphorylation of tau [12,13], possibly by activating kinases, including Raf/mitogen-activated protein kinase and inhibiting phosphatase-like protein phosphatase 2A (PP2A) [14,15,16]. Low micromolar levels of zinc can accelerate the fibrillization of human tau via bridging of Cys-291 and Cys-322 with in vitro study [17] and there is evidence that zinc binding directly regulates tau toxicity independent of tau hyperphosphorylation in a Drosophila tauopathy model expressing a human tau mutant [18]. Lastly, Ca^2+^ and Mg^2+^ may selectively induce the formation of NFT, whereas Al^3+^, Cu^2+^, and Fe^2+^/Fe^3+^ can bind to tau as shown by in vitro study [19], and immunohistochemical study in AD and control brain [20]. 

The antiparasitic clioquinol (CQ, iodochlorhydroxy-quin, 5-chloro-7-iodo-8-hydroxyquinoline, *M*_W_ = 305.5) is a metal protein attenuating compound (MPAC) with mild metal chelating function. CQ is hydrophobic, which enables it to effectively pass through the blood brain barrier (BBB) [21] to modulate Zn^2+^ and Cu^2+^ in the brain [22].

In the amyloid precursor protein (APP) 2576 transgenic AD mouse model, CQ enabled Zn^2+^ and Cu^2+^ to detach from SPs and reduce Aβ aggregation in cerebral cortices [23]. CQ also functions as an ionophore and can transfer Cu^2+^ from SPs to surrounding cells [24]. The increased Cu^2+^ concentration in the cytoplasm activates the matrix metalloproteinase which causes the degradation of Aβ [25]. 

As biometal imbalance also plays an important role in tau pathology, CQ may influence tau metabolism. In a small-scale clinical trial, CQ was administered at doses of 20 mg/day to 10 AD patients and 80 mg/day to another 10 AD patients for 21 days. Tau levels in cerebrospinal fluid (CSF) increased at day 7 and a decreased at day 21. Cognitive functions assessed by the Alzheimer’s Disease Assessment Scale (ADAS-Cog), including naming, instruction, and comprehensive scale improved after CQ treatment [26]. A randomized, double-blind, placebo-controlled pilot Phase II clinical trial [27] demonstrated that CQ had a significant protective effect against cognitive deterioration during the 36-week treatment period in more severely affected AD patients (baseline ADAS-cog subscale ≥25) [27]. The second generation of CQ, PBT2, was reported to be safe and beneficial for AD [28,29,30] and Huntington’s disease (HD) in phase II clinical trial. ATH434, an orally bioavailable 8-hydroxyquinazolin-4(3H)-one, which binds iron sufficiently [31], was approved as an orphan drug for multiple system atrophy in FDA.

The aim of our study was to clarify the effects of CQ on tau oligomerization using a cell culture model (M1C cells) that stably overexpresses wild type human 4R0N tau through a tetracycline-off (Tet-Off)-inducible mechanism [32,33,34,35,36,37].

## 2. Results

### 2.1. Up to 10 μM CQ Did Not Exhibit Cell Toxicity in M1C Cells

**Figure 1:**
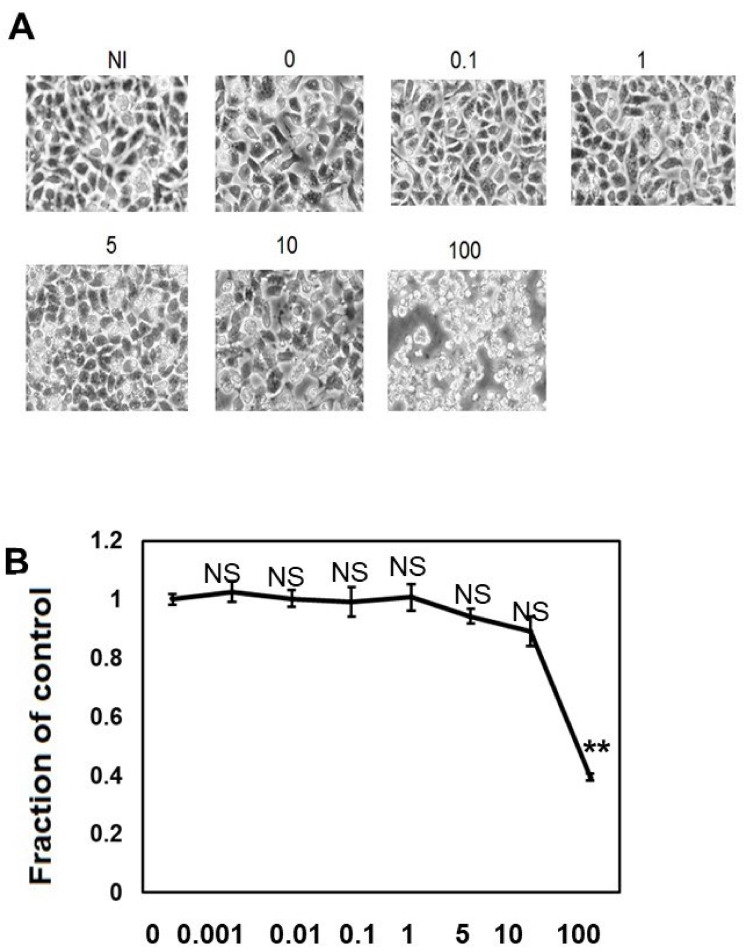
Clioquinol (CQ) does not affect cell viability at low doses. (**A**) Low dose (0.1 to 10 μM) of CQ did not cause morphological changes in M1C cells, but at a concentration of 100 µM, it was overtly toxic to cells. (**B**) The effects of CQ on M1C cell survival following TetOff induction of tau expression was examined by the ATP assay. The LD50 value obtained from the ATP assay was 80 µM CQ for M1C cells, and the reduced viability was significant at 100 µM. Data are the relative change compared with the control (1). NI: non-induced cells without CQ, *n* = 4, data are presented with ± SD, ** *p* < 0.01, versus vehicle control, as determined by ANOVA. N.S.: not significant.

We performed morphological analysis (Figure 1A) and ATP viability assay (Figure 1B) to elucidate the effects of different concentrations of CQ on M1C cell viability. When the cells were treated with CQ up to 10 μM, CQ did not cause morphological changes, and cell viability did not decrease, but 100 μM CQ caused morphological change and a 60.6% reduction in cell viability (Figure 1A,B). This demonstrated that CQ is not overtly toxic until a high dosage is used. 

### 2.2. CQ Decreased Cu^+^ Ions in the Cell

**Figure 2:**
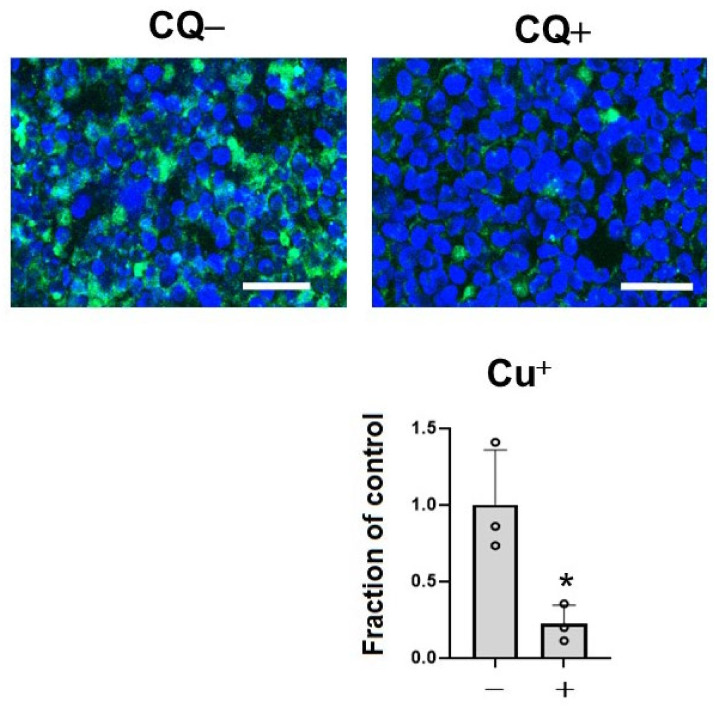
Clioquinol (CQ) decreased free Cu^+^ ions in M1C cells. M1C cells were exposed to 1 µM CQ or DMSO during the final day of induction. Cu^+^ iron deposition was detected by CopperGreen fluorescence probe of Cu^+^. Barr: 50 μm. At 1 μM, CQ significantly decreased Cu^+^ based on indicated measured intensity levels of CopperGreen signal. *n* = 3, data are presented with ± SD, * *p* < 0.05, versus vehicle control. The data followed a normal distribution and were analyzed by the student’s *t*-test.

To assess the effects of CQ as a metal chelator, we measured the level of Cu^+^, which is a dominant redox state of copper in an intracellular reducing environment by BioTracker CopperGreen Live Cell Dye. Treatment with 1 μM of CQ significantly decreased Cu^+^ ions in the cells (39.4% of control) (Figure 2). This suggested that CQ significantly decreased Cu^+^ levels in the cells.

### 2.3. CQ Decreased the Tau Levels Detected Using Non-Phospho Dependent Tau5 

**Figure 3:**
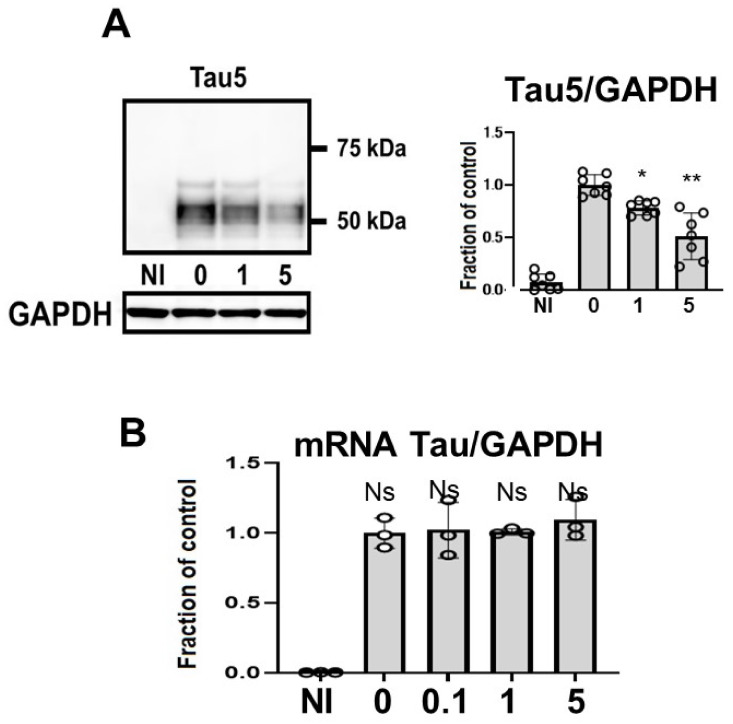
Clioquinol (CQ) decreased tau levels detected using non-phospho dependent Tau5 in M1C cells. M1C cells were exposed to CQ at 0, 1, or 5 μM during the final day of induction. (**A**) Lysates derived from cultures were immunoblotted with antibody against Tau5. CQ decreased the total amount of tau. *n* = 7, ** *p* <0.01, * *p* <0.05, Bar: ± SD. (**B**) CQ did not alter tau mRNA levels according to quantitative real-time PCR (qPCR). Data are the relative change compared with the control. *n* = 3, Bar: ± SD, ns—not significant. Data from Tau5/GAPDH, and mRNA Tau/GAPDH followed a normal distribution and were analyzed by one-way ANOVA followed by the Bonferroni post hoc test.

Tau levels detected using non-phospho dependent Tau5 decreased in a dose-dependent manner (Figure 3A). Induced M1C cells exhibited expression of tau protein that was detected at molecular weights between 52–68 kDa. CQ decreased the levels of tau levels detected using non-phospho dependent Tau5, with significant reduction by 5 μM CQ (−51.3 ± 22.2%). CQ did not alter tau mRNA levels according to qPCR (Figure 3B).

### 2.4. CQ Decreased Phosphorylated Tau

**Figure 4:**
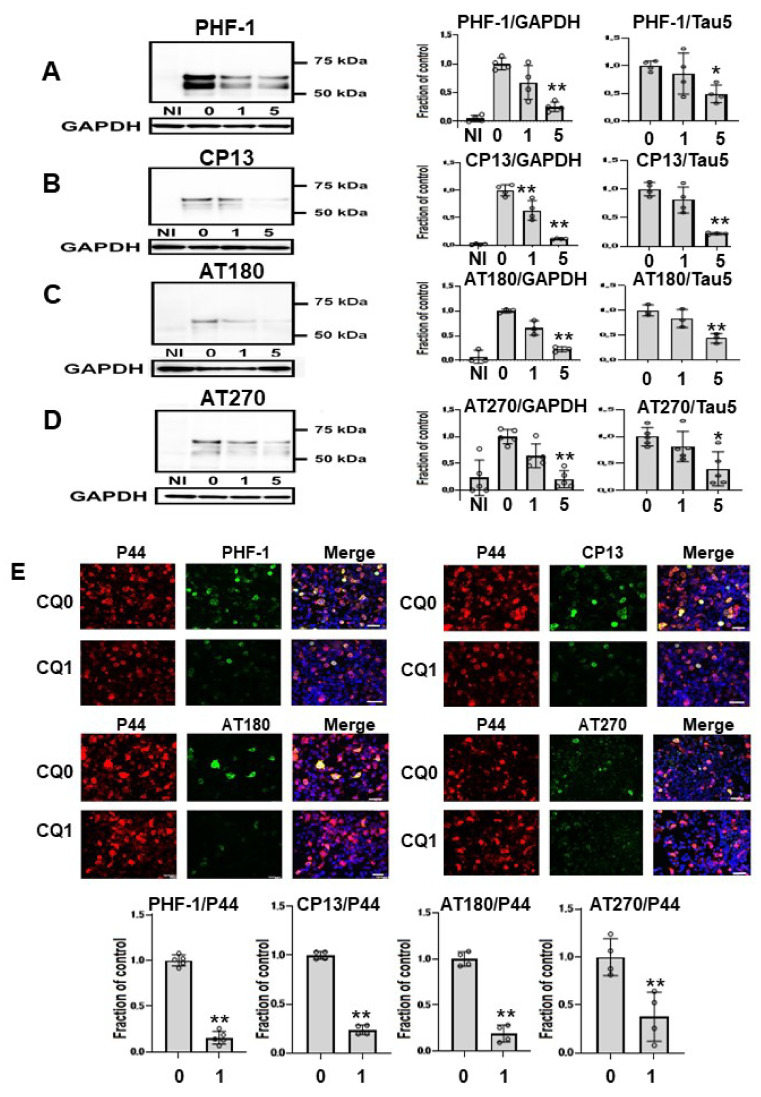
Phosphorylated tau was decreased by clioquinol (CQ). Phosphorylated tau was significantly decreased by 5 μM CQ, detected by PHF-1(*n* = 4) (**A**), CP13 (*n* = 4) (**B**), AT180 (*n* = 3) (**C**), and AT270 (*n* = 5) (**D**). Phosphorylated tau detected by CP13 or AT 180 was also significantly decreased by 1 μM CQ. Bar: ± SD, ** *p* < 0.01, * *p* < 0.05. The ratio of phosphorylated tau to tau levels detected using non-phospho dependent Tau5 with 5 μM CQ also decreased based on antibodies against PHF-1, CP13, AT180, and AT270. Immunocytochemical study revealed that CQ (1 μM) decreased phosphorylated tau. The ratio of phosphorylated tau to tau levels detected using non-phospho dependent Tau5 with 1 μM CQ also decreased based on antibodies against PHF-1 (*n* = 5), CP13 (*n* = 4), AT180 (*n* = 4), and AT270 (*n* = 4). CQ 0: DMSO-treated cells, CQ1: 1 μM CQ-treated cells.** *p* < 0.01, * *p* < 0.05, Bar: 50 μm (**E**). Data from PHF-1/GAPDH, CP13/GAPDH, AT180/GAPDH, and AT270/GAPDH followed a normal distribution and were analyzed by one-way ANOVA followed by the Bonferroni post hoc test, and the data from PHF-1/P44, CP13/P44, AT180/P44, and AT270/P44 followed a normal distribution and were analyzed by the Student’s *t*-test.

Next, we assessed effects of CQ on tau phosphorylation using four phospho-tau antibodies (Figure 4A–D), and membranes were then reprobed for GAPDH. Treatment of cells with 1 μM CQ significantly decreased phosphorylated tau detected by CP13 or AT 180, whereas there was no significant difference in phosphorylated tau species detected by PHF-1, or AT270. In cultures treated with 5 μM CQ, the level of PHF-1-positive tau was 25.4 ± 8.2% of vehicle controls, that of CP13 was 11.4 ± 1.0% of vehicle controls, that of AT180 was 22.6 ± 4.9% of vehicle controls, and that of AT270 was 22.2 ± 18.2% of vehicle controls. The ratio of phosphorylated tau to tau levels detected using non-phospho dependent Tau5 significantly decreased after 5 μM CQ treatment. Similarly, significant decreases were detected with PHF-1, CP13, AT180, and AT270 phospho-tau antibodies when normalized to tau levels detected using non-phospho dependent Tau5. We also used immunocytochemical analysis to evaluate the effects of CQ on tau phosphorylation. PHF-1, CP13, AT180, and AT270 were selected as antibodies for phosphorylated tau, and P44 detects tau levels detected using non-phospho dependent Tau5. In cultures treated with 1 μM CQ, the ratio of PHF-1, CP13, AT180, and AT270-positive cells to P44-positive cells decreased (Figure 4E). This suggested that CQ decreases phosphorylated tau.

**Figure 5:**
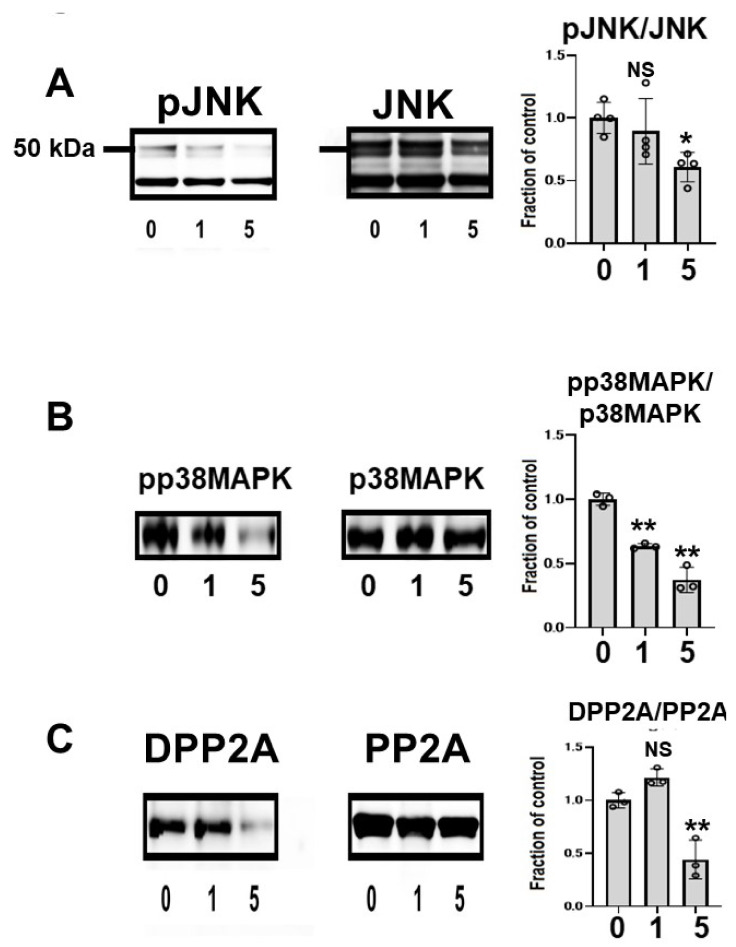
Clioquinol (CQ) inactivated c-Jun N-terminal kinase (JNK) and activated protein phosphatase 2A (PP2A) in M1C cells. (**A**) Immunoblotting showed that CQ decreased phosphorylated JNK (pJNK), the active form of the kinase. *n* = 4. (**B**) CQ treatment also decreased the active, phosphorylated form of p38 mitogen activated protein kinase (p38 MAPK) (pp38 MAPK). *n* = 3. (**C**) CQ treatment decreased demethylated PP2A, which represents the inactive form of PP2A. *n* = 3 (**C**). 0:0 μM CQ-treated cells, 1:1 μM CQ-treated cells, and 5:5 μM CQ-treated cells. Bar: ± SD, ** *p* < 0.01, * *p* < 0.05. Data from pJNK/JNK, DPP2A/PP2A, pp38 MAPK/p38 MAPK followed a normal distribution and were analyzed by one-way ANOVA followed by the Bonferroni post hoc test.

To clarify the effects of CQ on tau kinase, we measured the levels of c-Jun N-terminal kinase (JNK) and phosphorylated JNK (pJNK) in lysates. Total JNK was unchanged across treated cells, but pJNK was lower in CQ-treated cultures than in controls. In cultures treated with 5 μM CQ, the pJNK/JNK levels were 60.7% of controls (Figure 5A). p38 mitogen-activated protein kinase (p38 MAPK) was also examined after CQ treatment. CQ decreased the phosphorylation level of p38 MAPK (pp38 MAPK), whereas the total amount of p38 MAPK did not differ. In cultures treated with 1 or 5 μM CQ, the pp38MAPK/p38MAPK levels were 63.5% and 37.3% of controls, respectively. This suggests that p38 MAPK was inactivated by CQ (Figure 5B); therefore, CQ can inactivate tau kinases.

The subunit of PP2A was analyzed using two different antibodies, anti-demethylated PP2A (DPP2A) and anti-total PP2A. DPP2A, the inactive form of PP2A, was decreased by CQ treatment (Figure 5B) [38,39], suggesting that CQ activates tau phosphatase.

### 2.5. CQ Decreased Caspase Cleaved Tau

**Figure 6:**
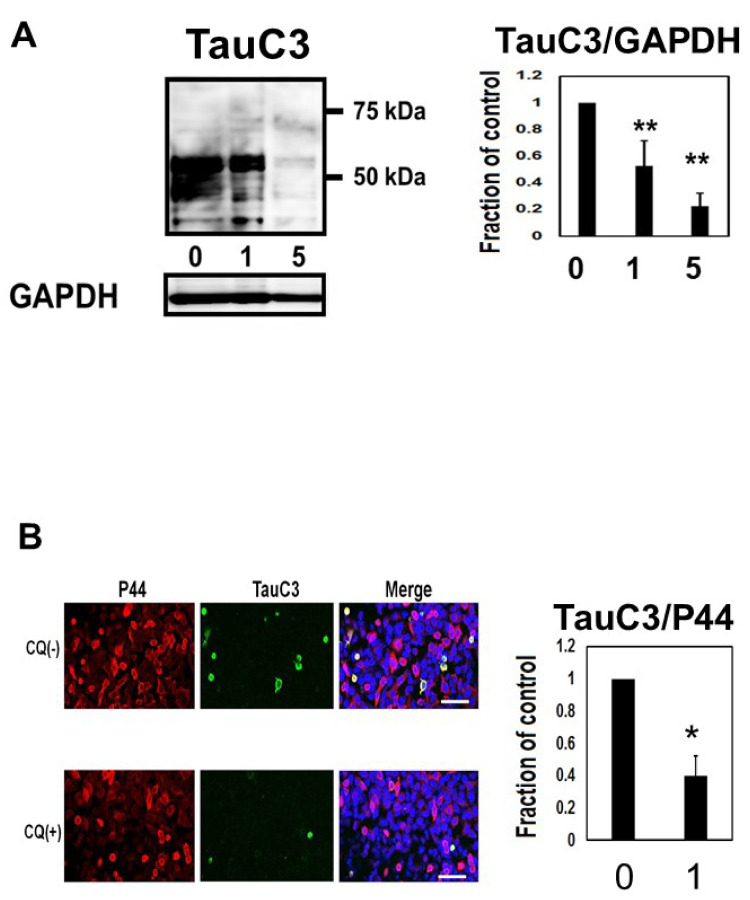
Clioquinol (CQ) decreased caspase cleaved tau in M1C cells. (**A**) CQ decreased caspase cleaved tau on Western blot analysis. NI: non induced, 0: vehicle-treated cells, 1, and 5:1, and 5 μM CQ-treated cells. *n* = 4, Bar: ± SD, ** *p* < 0.01. (**B**) Analysis of TauC3 immunofluorescence signal (ratio to signal from P44, a non-phospho dependent tau antibody) showed a significant reduction following 1 μM of CQ treatment. 0: vehicle-treated cells, 1:1 μM CQ-treated cells. *n* = 4, Bar: 50 μm, * *p* < 0.05. TauC3/GAPDH followed a normal distribution and was analyzed by one-way ANOVA followed by the Bonferroni post hoc test, and the data from TOC1/P44 followed a normal distribution and were analyzed by the Student’s *t*-test.

We assayed whether CQ treatment alters the level of caspase cleaved tau using TauC3 Western blot analysis (Figure 6A). In cultures treated with 1 or 5 μM CQ, TauC3 decreased by 77.4 ± 9.8%. Immunocytochemical study also revealed the reduction of TauC3-positive cells (Figure 6B), suggesting that CQ decreases C-terminal truncated, toxic tau species.

### 2.6. CQ Decreases Tau Oligomers in M1C cells

**Figure 7:**
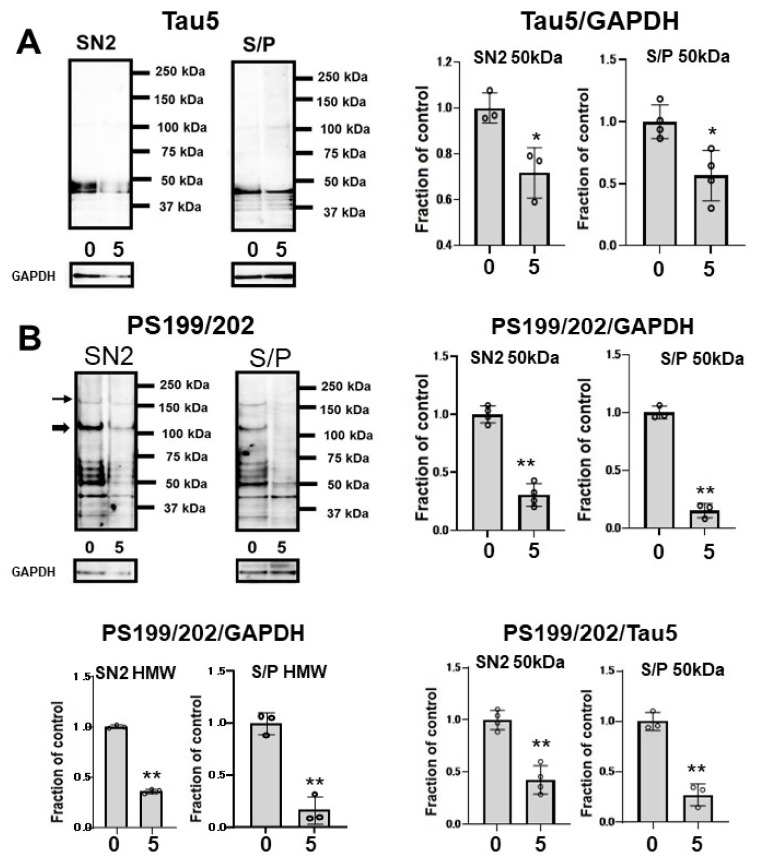
Clioquinol (CQ) suppresses the accumulation of sarkosyl-insoluble tau in M1C cells. Fractionational analysis was performed following CQ treatment, and cells were separated into SN1 (Tris soluble), SN2 (Tris insoluble-sarkosyl-soluble), and S/P (sarkosyl-insoluble pellet) fractions. Each fraction was then analyzed by Western blot using Tau5 (**A**), and PS199/202 antibodies (**B**). The amount of tau was decreased in S/P fractions obtained from cells treated with CQ. The ratio of phosphorylated tau vs tau detected by non-phospho dependent antibody Tau5 by 5 μM CQ also decreased, which was detected by antibodies against PS199/202. 0: 0 μM CQ, 5: 5 μM CQ. *n* = 4, * *p* < 0.05, ** *p* < 0.01, NS: not significant, Bar ± SD. All the data followed a normal distribution and were analyzed by the Student’s *t*-test.

To assess whether CQ influences tau oligomers, M1C cells were induced to express tau for 5 days and exposed to 5 μM of CQ on Day 4. SN1 (buffer soluble), SN2 (tris insoluble sarkosyl-soluble), and S/P (sarkosyl-insoluble pellet) fractions were assayed using Tau5 and pS199/202 antibodies in Western blotting. In the SN2 and S/P fractions, tau detected by non-phospho dependent antibody Tau5 decreased (Figure 7A). Phosphorylated high-molecular weight (HMW) tau species (120 kDa and 160 kDa) and tau around 50 kDa detected by pS199/202 antibody decreased in the SN2 fraction following treatment with 5 μM of CQ. In the S/P fraction, HMW and ~50-kDa tau bands also decreased after 5 μM CQ treatment (Figure 7B). 

**Figure 8:**
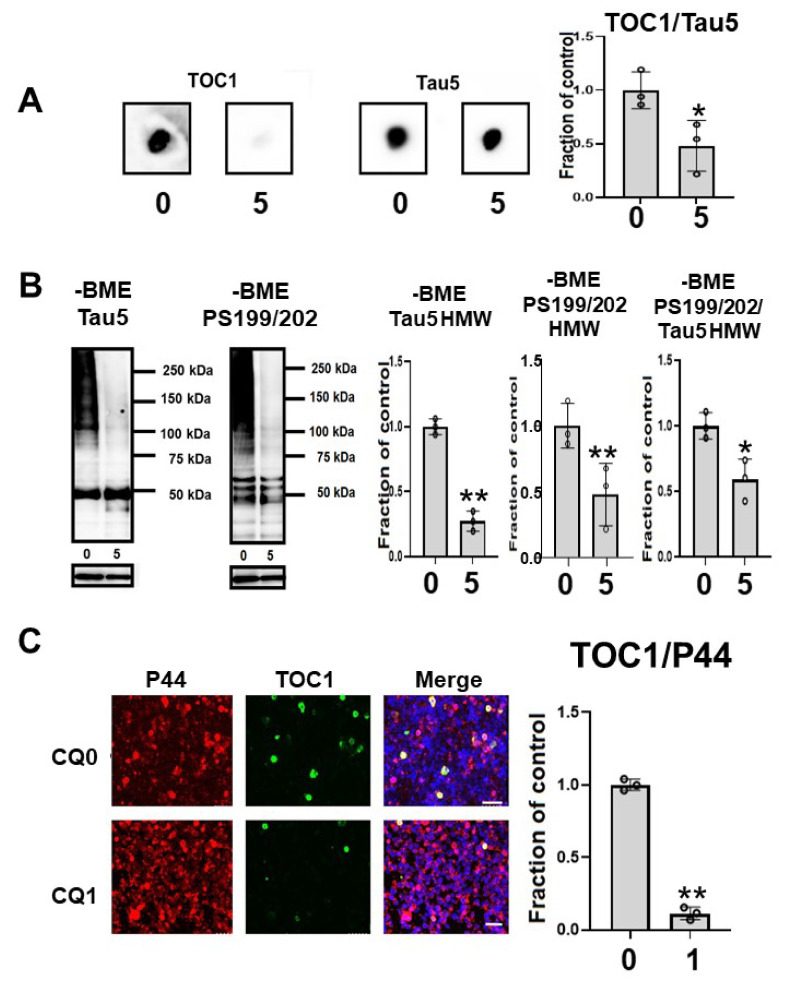
Clioquinol (CQ) decreased oligomeric tau in M1C cells. (**A**) Dot blot analysis of lysate demonstrated the reduction of oligomeric tau by 5 μM CQ detected by TOC1, which is a tau oligomer-specific antibody. *n* = 3, * *p* <0.05, Bar: ± SD (**B**) CQ treatment decreased high molecular weight smeared tau in non-reducing condition on Western blotting. The ratio of phosphorylated tau to non-phospho dependent antibody Tau5 with 5 μM of CQ also decreased, which was detected by antibodies against PS199/202. *n* = 3, * *p* <0.05, Bar: ± SD (**C**) Analysis of oligomeric TOC1 immunofluorescence signal (ratio to signal from P44, a non-phospho dependent tau antibody) showed a reduction following 1 μM CQ treatment. Bar 50 μm. *n* = 3, ** *p* < 0.01, Bar ± SD. Dot blot analysis of TOC1, high-molecular weight smear tau in non-reducing conditions, and TOC1 immunofluorescence analysis; the data followed a normal distribution and were analyzed by the Student’s *t*-test.

Oligomeric tau species, as indicated by the oligomer specific TOC1 antibody reactivity in dot blots, were significantly decreased by CQ treatment (Figure 8A). In the immunocytochemical study, TOC1-positive cells were decreased by 75.0% following CQ treatment (Figure 8C). This suggested that CQ decreases oligomeric tau species.

### 2.7. CQ Upregulated Autophagy and Activated Proteasomes

**Figure 9:**
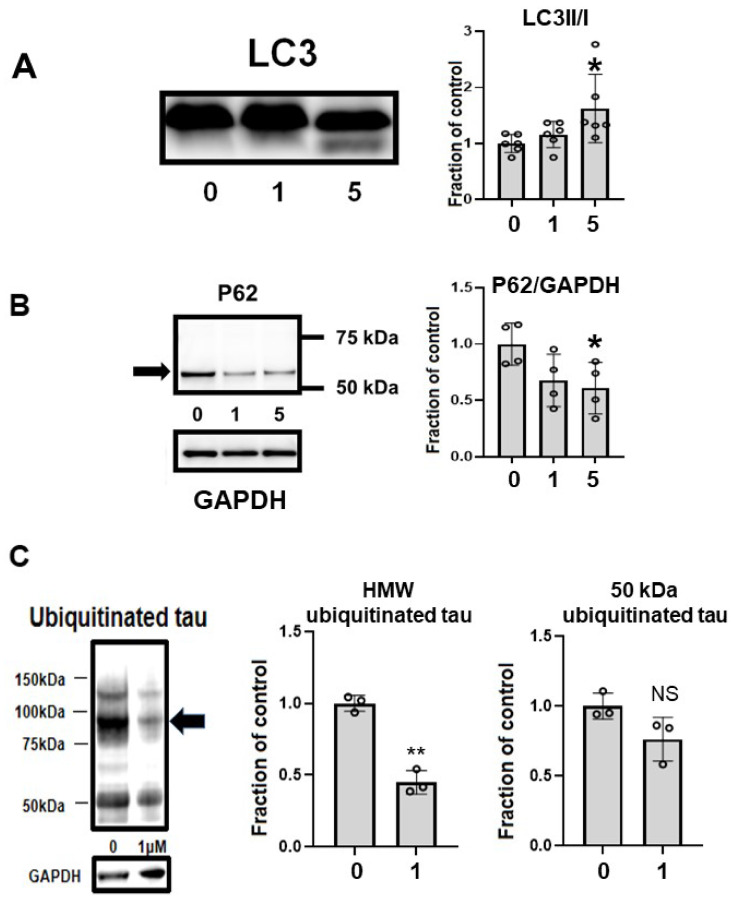
Clioquinol (CQ) upregulated autophagy and activated proteasomes. (**A**) CQ upregulated autophagy in MC1 cells based on the increase in LC3-II, a marker of autophagy, after treatment with 5 μM CQ. *n* = 6, * *p* < 0.05, Bar ± SD. (**B**) Another marker of autophagy, p62, was decreased after CQ treatment. *n* = 4, * *p* < 0.05, Bar ± SD. This suggested that autophagy was upregulated. (**C**) CQ decreased the amount of ubiquitinated tau protein, suggesting that the proteasomal system was upregulated, especially for high-molecular weight species (arrow). *n* = 4, ** *p* < 0.01, Bar ± SD. LC3II/I followed a normal distribution and was analyzed by one-way ANOVA followed by the Bonferroni post hoc test. The high-molecular weight and 50-kDa ubiquitinated tau data followed a normal distribution and were analyzed by the Student’s *t*-test.

CQ upregulated autophagy in MC1 cells. LC3-II, a marker of autophagy, was upregulated by 5 μM CQ treatment (Figure 9A). Another marker of autophagy, p62, was decreased after CQ treatment (Figure 9B), suggesting that autophagy was upregulated. CQ treatment decreased the level of ubiquitinated tau, especially in HMW ubiquitinated tau (Figure 9C). This suggested that CQ activated the proteasomal system. 

### 2.8. CQ Decreased Endogenous Tau 

**Figure 10:**
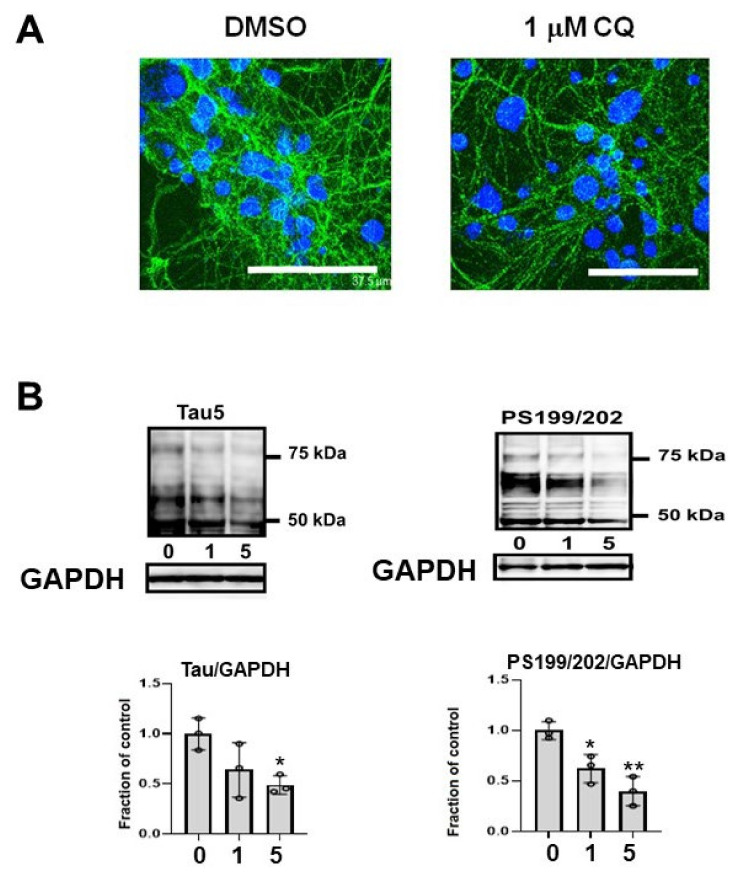
Endogenous tau also decreased after clioquinol (CQ) treatment. (**A**) In primary cortical neuronal cultures, 1 μM CQ decreased phosphorylated tau (CP13) (Bar: 37.5 μm). (**B**) Endogenous tau in non-induced M1C cells was decreased by CQ. Tau detected by non-phospho dependent tau antibody (Tau5) was decreased by 5 μM CQ treatment and phosphorylated tau (PS199/202) levels were decreased by 1 or 5 μM CQ treatment. *n* = 3, ** *p* < 0.01, * *p* < 0.05, Bar ± SD.

In the cultured mouse primary cortical neurons, phosphorylated endogenous tau (CP13) decreased (Figure 10A). Endogenous tau in non-induced M1C cells was decreased by CQ treatment. Tau detected by non-phospho dependent tau antibody (Tau5) and phosphorylated tau (PS199/202) levels were decreased by CQ treatment (Figure 10B). 

## 3. Discussion 

We explored whether the metal-protein attenuating compound CQ decreased multiple forms of tau protein including phosphorylated, C-terminal truncated and oligomeric tau species in a cell culture model of tauopathy [32,33,34,35,36,37]. As the tau degradation mechanisms, CQ activated autophagy and proteasomal systems, suggesting that they play a role in reducing tau levels. The inactivation of tau kinases, including p38 MAPK and JNK, and activation of the tau phosphatase PP2A following CQ treatment also suggested it decreases pTau species in cells.

After CQ treatment, deposition of Cu^+^ in the cell was markedly decreased (Figure 2). In an epidemiological study, high dietary intake of copper in elderly Japanese women was associated with the severity of subjective forgetfulness [40]. PHF-1, CP13, AT180, and AT270-positive phosphorylated tau decreased, suggesting that phosphorylation of tau was suppressed at Ser394/404, Thr231, Thr181, Thr205, and Ser202. On immunocytochemical analysis, the ratio of PHF-1, CP13, AT180, and AT270-positive (phosphorylated tau-positive cells) cells to P44-positive cells (non-phospho dependent tau antibody-positive cells) decreased after treatment with 1 μM CQ (Figure 4E). The CQ decreased endogenous tau, as indicated by non-phospho dependent tau antibody Tau5 assays, and phosphorylated tau (PS199/202) in non-induced M1C cells. It also decreased endogenous phosphorylated tau in cultured mouse primary cortical neurons. These results were consistent with the previous study using human tau transgenic mice (B6.Cg-Mapttm1(GFP)KltTg(MAPT) 8cPdav/J). After being treated with 30 mg/kg/day of CQ for 5 weeks, the tau phosphorylation level in the brain decreased including sites at Ser396, Thr205, Ser214, and Ser404 [16]. In addition to hyperphosphorylation, truncation is another important abnormal posttranslational modification for tau [41,42]. TauC3 antibody detects the C-terminal truncation of tau at D421 [43]. In the present study, TauC3-positive truncated tau decreased after CQ treatment (Figure 6), which suggests that CQ represses the truncation of tau or increases its turnover.

We also investigated the mechanism underlying the suppression of tau phosphorylation and truncation. We found that CQ treatment did not influence the total amount of JNK (Figure 5) but decreased its active form pJNK. JNK is an important kinase in tauopathy. Increased expression of JNK was found in brain homogenates from tauopathy patients [44]. In neurons and glial cells that contain hyperphosphorylated tau, and in dystrophic neurites surrounding SPs, the activity of JNK increases. Furthermore, JNK is known as an important pathway of tau phosphorylation [45,46,47,48]. PP2A activity was upregulated by CQ treatment (Figure 5C), and although Thr 205, 212, Ser 214, and 262 are described as the most favorable sites, CP13-positive phosphorylated tau (PThr202/PSer205) was most decreased in our study.

NFTs may not be toxic; however, malformed oligomeric tau may be associated with neuronal dysfunction before NFT formation [49]. Tau oligomers, the intermediate form of tau pathology was reported to be neurotoxic in both in vivo and in vitro experiments [50]. Among different methods for investigating insoluble tau oligomers, sarkosyl extraction is the standard protocol [51]. There is a growing consensus that sarkosyl-insoluble tau correlates with the pathological features of tauopathy. In our study, we examined the effects of CQ on tau oligomers in both the sarkosyl-soluble fraction and sarkosyl-insoluble fraction. In 5 μM CQ-treated cultures, Tau5, AT180, and AT270-positive oligomers decreased in the sarkosyl-insoluble fraction; therefore, both non-phospho dependent tau antibody-Tau5 positive tau oligomers and phosphorylated tau oligomers were decreased. Indeed, TOC1, which specifically detects oligomeric tau [35,36,37,52,53], was decreased by CQ treatment on dot blot (Figure 8A), Western blot analysis (Figure 8B), and immunocytochemical study (Figure 8C). 

In 1970, CQ was withdrawn from the market in Japan because it was considered to cause subacute myelo-optico-neuropathy (SMON) [54]. SMON is a disease of subacute onset characterized by sensory and motor disorders in the lower limbs, visual impairment, and abdominal symptoms. The mechanism of SMON was hypothesized to be associated with abnormal metabolism of vitamin B_12_, as a study in mice demonstrated that CQ decreased the serum vitamin B_12_ concentration [55]. In a clinical trial, vitamin B_12_ supplementation was started before CQ treatment for 3 weeks and no severe side effects were noted [26]. In our study, low concentrations of CQ (0.001 μM to 10 μM) exhibited no cytotoxicity, however, higher doses of CQ caused cytotoxicity, with 100 μM CQ significantly reducing cell viability. 

Previous studies reported that CQ alleviates Aβ toxicity in mouse models of AD [23,30]. A derivative of CQ, ATH434, is an orally bioavailable 8-hydroxyquinazolin-4(3H)-one that is currently FDA-approved for the treatment of multiple system atrophy, which is a synucleinopathy [31]. 

**Figure 11:**
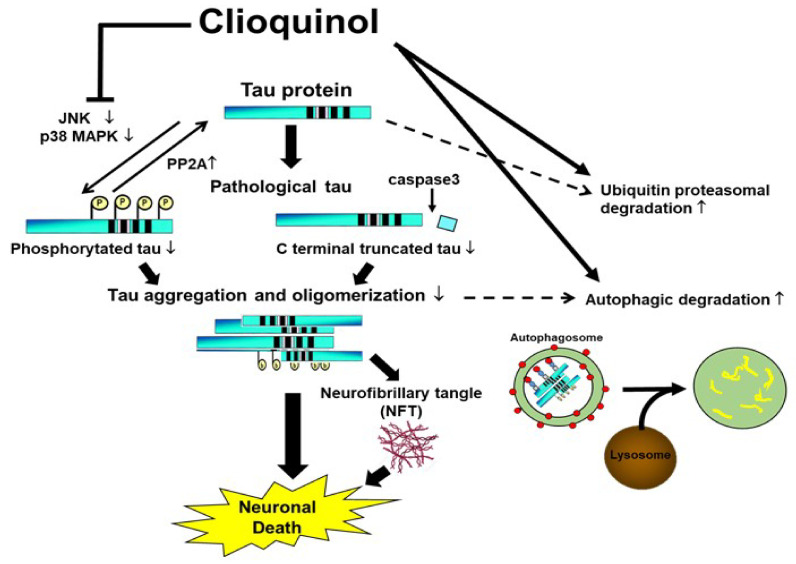
Proposed mechanisms by which clioquinol (CQ) decreases pathological tau species. CQ inactivated tau kinases, including JNK and p38 MAPK, while also increasing tau phosphatase activation. CQ may also decrease the C-terminal truncation of tau and formation of tau oligomers, which are 2 forms of tau linked to toxicity. It also upregulated autophagy and proteasome system activity, possibly leading to an overall reduction in tau, including pathologically modified tau species.↑: upregulation, ↓:downregulation.

Collectively, our study suggests that CQ therapy may help suppress pathological changes in tau protein through three routes. First, CQ may decrease phosphorylation through inactivation of tau kinases such as JNK and pp38 MAPK, and activation of tau phosphatases such as PP2A. Second, CQ may activate the autophagy and proteasomal systems to promote tau turnover in cells (Figure 11). Lastly, CQ decreases the formation of insoluble tau oligomers. Thus, CQ may represent a potentially novel therapeutic treatment for AD.

## 4. Materials and Methods

### 4.1. Materials and Antibodies 

**Figure 12:**
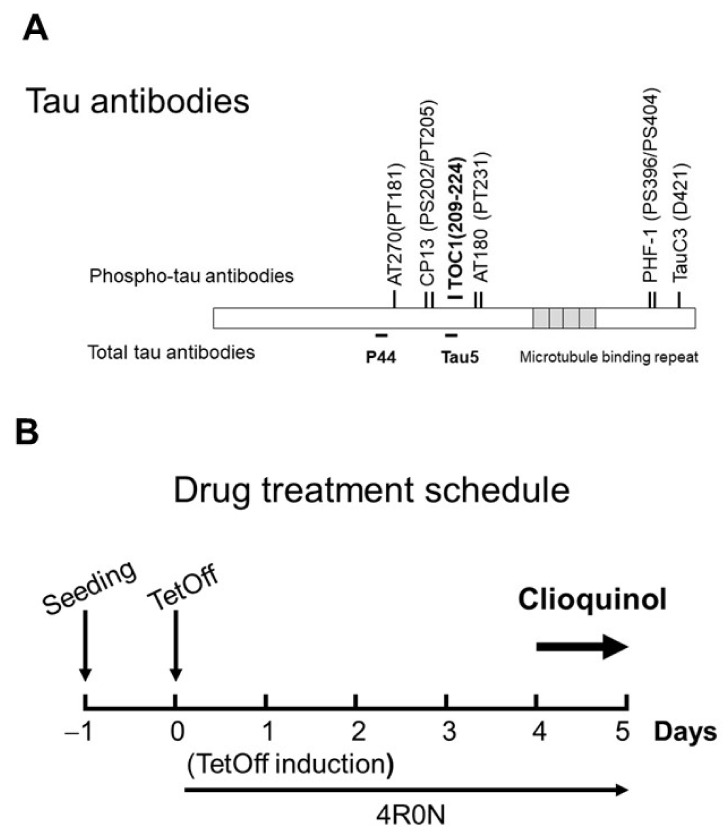
Tau antibodies used in this study, and the experimental timeline. (**A**) Tau antibodies used, and distinct epitopes recognized by different antibodies. (**B**) Schedule of tetracycline off (TetOff) induction. On Day 0, TetOff induction was started by reducing the concentration of tetracycline in the medium from 2000 to 1 ng/mL. On Day 4, clioquinol treatment was initiated. On Day 5, cells were harvested.

Tissue culture ware was obtained from BD Biosciences (Franklin Lakes, NJ, USA). CQ was obtained from Sigma-Aldrich (Cat Number #33931, St. Louis, MO, USA). Other chemicals were obtained from Sigma unless otherwise indicated. Tau antibodies used in this study (Figure 12A) were extensively used in previous studies [32,34,35,36,37,41,52,56]. The monoclonal antibody Tau5 was obtained from Invitrogen (Carlsbad, CA, USA); PHF-1, and CP13 were obtained from Dr. Peter Davies (The Feinstein Institute for Medical Research, Manhasset, NY, USA). AT180 and AT270 were from Thermo (catalog #MN1040, #MN1050, Rockford, IL, USA). TauC3 was obtained from Santa Cruz (catalog #sc-32240, Santa Cruz, CA, USA); Tau Oligomeric Complex I antibody (TOC1, provided by Kanaan Lab) that recognizes tau dimers and high-ordered oligomers [35,52,53]. Glyceraldehyde 3-phosphate dehydrogenase (GAPDH) was obtained from Millipore (catalog #MAB374, Billerica, MA, USA). Monoclonal antibodies against phosphorylated tau protein, JNK, pJNK (Thr183/Tyr185), PP2A, and DPP2A were purchased from Cell Signaling (catalog #9258, #4668, #sc-80665, and #sc-13601, Danvers, MA, USA). Polyclonal antibodies p38 MAPK and pp38 MAPK (Thr180/Tyr182) were from Cell Signaling: catalog #92112, and #9211). Antibodies were used at the following dilutions: Tau5 (1:1,000), PHF-1 (1:200), CP13 (1:200), TauC3 (1:1000), AT180 (1:1000), AT270 (1:1000), TOC1 (1:1000), anti-GAPDH (1:2000), PP2A (1:1000), DPP2A (1;1000), JNK (1:1000), pJNK (1:1000), p38 MAPK (1:1000), and pp38 MAPK (1:1000).

### 4.2. Cell Culture 

M1C cells were seeded at 1.5–2 × 10^6^ cells per plate in Dulbecco’s modified Eagle’s medium which contained tetracycline (Tet) (2 μg/mL), 10% fetal bovine serum, and G418 (400 g/mL: Life Technologies, Gaithersburg, MD, USA). Two days after seeding, TetOff induction was started by replacing spent medium with fresh medium containing 1 ng/mL of Tet to elicit tau expression. At that time, the confluency was about 50%. On Day 4 of TetOff induction, replica cultures of M1C cells were exposed to CQ (0, 1, and 5 μM) (about 70%–80% of confluency) and at the end of Day 5 (about 95–100% of confluency), cells were harvested for tau monomer experiments (Figure 12B). In the tau oligomer experiments, we used CQ at 0 and 5 μM (Figure 8A). Dimethyl sulfoxide (DMSO, at 0.01%), the vehicle used to dilute CQ, was used to treat the control cultures. Supernatants of lysates from cultures were analyzed by using Western blot analysis with the primary antibody against Tau5. Thereafter, GAPDH antibody was applied to confirm equal loading among lanes.

Isolated primary neurons from Slc: ICR mice at embryonic day 16 (E16) were seeded on Lab-Tek chambered cover glasses precoated with polyethyleneimine (PEI) at a density of 5 × 10^4^ per well. Cultured neurons were maintained in serum-free Neurobasal medium (Invitrogen) with 5 μM Ara C to prevent non-neuronal cell proliferation [34,35,37,57]. All experiments in the present study were carried out using pure neuronal cells (>95% neuronal). The purity of the cells was estimated by staining with neuron specific anti-NeuN (Millipore). Animal procedures were approved by the University of Fukui.

Cultured M1C cells grown in 24 well plates at a density of 2.6–3.5 cells x 10^3^/cm^2^ were subjected to morphological studies with or without CQ treatment after the TetOff induction. Phase contrast imaging of M1C cells was performed using an inverted microscope (IX-70: Olympus, Tokyo, Japan) equipped with a digital camera (DP-70: Olympus) and the images were processed using Adobe Photoshop Elements Version 13 (Adobe, San Jose, CA, USA) for publication. 

The Cell Titer-Glo assay (Promega, Cat#G7570), which utilizes adenosine triphosphate (ATP) to assay cell viability, was used to measure the extent of cell toxicity according to the manufacturer’s instructions [34]. Briefly, M1C cells with TetOff induction or mouse primary cortical neurons were cultured in 384 well plates with or without CQ. Twenty-five μL of mixed reagents was added in each well and luminescence was measured by SpectraMax M5 microplate reader (Molecular Devices, Sunnyvale, CA). Each experimental group was repeated in four wells.

### 4.3. Fractionation of Cell Lysates

After harvest, M1C cells were homogenized in Tris buffer containing phosphatase and protease inhibitors (30 mM β-glycerophosphate, 30 mM sodium fluoride, and protease inhibitor cocktail; Roche, Mannheim, Germany), 1 mM ethylene glycol tetraacetic acid (EGTA), and 1 mM ethylenediamine tetraacetic acid (EDTA). Homogenates were centrifuged at 1500× *g* for 15 min. Based on solubility in Tris buffer or 2% sarkosyl, supernatants were further fractionated to generate SN1, SN2, and S/P fractions, as reported previously [32,34,35,36,37,58]. The SN1 fraction was the supernatant obtained from centrifugation of lysates at 45,000 g for 15 min at 4 °C. The pellet was resuspended in buffer containing 10% sucrose, 0.8 M NaCl, 10 mM Tris/HCl (pH 7.4), 1 mM EGTA, protease inhibitor cocktail, and 1% sarkosyl and centrifuged at 150,000 x g for 15 min. The supernatant was SN2 and the sarkosyl-insoluble pellet was S/P. SN1, SN2, and S/P resuspended in Tris buffer was immediately used for immunoblotting or stored at −20 °C.

### 4.4. Western Blotting

Cell lysates or fractionated preparations were mixed with Laemmli sample buffer with or without 1% β-mercaptoethanol (βME) for immunoblotting. Protein concentrations of cell lysates were measured by the bicinchoninic acid assay (Thermo Scientific, Rockford, IL, USA) [34,35]. Equal amounts of proteins (10 μg, corresponding to 0.1–0.2×10^5^ cells per lane) were loaded and electrophoresed on SDS-polyacrylamide gels, and subsequently transferred onto polyvinylidene difluoride (PVDF) membranes (Immobilon P; Millipore). Membranes were immersed in 1% gelatin in Tris-buffered saline containing 0.1% Tween 20 (TBS-T) for blocking for 1 h at room temperature (RT). After washing with TBS-T, membranes were incubated with monoclonal antibodies against Tau5, CP13, AT180, AT270, TauC3, and JNK for 1 h at RT. After the third wash, membranes were incubated with horseradish peroxidase-conjugated secondary antibodies including sheep anti-mouse immunoglobulin G (IgG) and goat anti-rabbit IgG for 30 min at RT. After a final wash, immunoreactive proteins were visualized using an enhanced chemiluminescence (ECL) Prime system (Amersham, Buckinghamshire, UK) [34,35,36,37]. Immunoreactivity was measured on captured images directly by Image Quant LAS 4000mini (GE Healthcare UK, Amersham Place, Buckinghamshire, UK), and ImageJ software (version 1.51, NIH, Bethesda, MD, USA) was used for densitometry measurements of immunoreactive bands (NIH, Bethesda, MA, USA). 

### 4.5. Immunocytochemical Study

Cells were cultured on Lab-Tek chambered coverglass at a density (5×10^5^/well) 1 day before TetOff induction. After 5 days of induction, cultures were treated with CQ (1 μM) or DMSO vehicle for 24 h. After washing with phosphate-buffered saline (PBS), cells were permeabilized with 0.25% Triton X-100/PBS and exposed to methanol at 20 °C prior to fixation in 2% paraformaldehyde/PBS. Fixed samples were then washed with PBS and blocked with PBS containing 3% goat serum. Then, samples were incubated with the primary antibodies against P44 and PHF-1, CP13, AT180, AT270, TauC3, or TOC1 followed by Alexa 594 anti-rabbit IgG (Molecular Probes, Eugene, OR, USA) and Alexa 488 anti-mouse IgG (Molecular Probes). A confocal fluorescence microscope (TCS SP II; Leica, Heidelberg, Germany) was used to visualize immunoreactivity.

### 4.6. Cu^+^ Visualization in the Cells

Cells were cultured on Lab-Tek chambered coverglass at a density (5×10^5^/well) 1 day before TetOff induction. After 5 days of induction, cultures were treated with CQ (1 μM) or DMSO for 24 h. The medium was replaced by that containing 5 μM CopperGreen (GORYO Chemical, Sapporo, Japan), which is a fluorescent probe to detect Cu^+^, for 3 h. After washing with PBS, cells were mounted in Vectashield mounting medium with DAPI (H-1200) (Vector Laboratories, Burlingame, CA, USA). A confocal fluorescence microscope (FV1200, X83, Olympus, Japan) was used to visualize immunoreactivity. To analyze the immunoreactivity, the number of positively stained cells was manually counted [36,37].

### 4.7. mRNA Expression 

Quantitative real-time PCR (qPCR) was performed according to our previous report [34,35]. Briefly, 20 ng cDNA per sample in a 20 μL reaction volume was used with the StepOnePlus Realtime PCR System. Relative gene expression was calculated by the ΔCT method [59]. All experiments were run in triplicate. 

### 4.8. Immunoprecipitation of Ubiquitinated Tau 

After normalization of protein levels, tau in the lysate was immunoprecipitated with the Tau5 antibody that recognizes non-phospho dependent antibody Tau5 as described previously [36,60]. The resulting immunoprecipitates were transferred onto PVDF membranes and probed with ubiquitin antibody (P4D1) to detect ubiquitinated tau protein [36].

### 4.9. Statistical Analysis 

All values are shown as the mean ± SD in the figures. We assessed the differences among more than two groups using one-way analysis of variance followed by the Bonferroni post hoc test if the data had a normal distribution and with the Kruskal–Wallis test followed by Dunn’s post hoc test if they deviated from the normal distribution. The differences between two groups were evaluated by the student’s t-test if the data had a normal distribution and by the Mann–Whitney U test if they deviated from the normal distribution (IBM SPSS Statistics, version 26, IBM Corp, Armonk, NY, USA), with p< 0.05 considered significant. 

## Data Availability

The data that supports the findings of this study are available within the article.

## Abbreviations

AD	Alzheimer’s disease
CQ	clioquinol
JNK	c-Jun N terminal kinase
P38MAPK	p38 MAP Kinase
NFT	neurofibrillary tangle
CC3	cleaved caspase3
PP2A	protein phosphatase 2A
GSK3β	glycogen synthase kinase 3β

## References

[1] Ihara Y., Nukina N., Miura R., Ogawara M. (1986). Phosphorylated tau protein is integrated into paired helical filaments in Alzheimer’s disease. J. Biochem..

[2] Bush A.I. (2003). The metallobiology of Alzheimer’s disease. Trends Neurosci..

[3] Hager K., Baseman A.S., Nye J.S., Brashear H.R., Han J., Sano M., Davis B., Richards H.M. (2014). Effects of galantamine in a 2-year, randomized, placebo-controlled study in Alzheimer’s disease. Neuropsychiatr. Dis. Treat..

[4] Cummings J.L., Schneider E., Tariot P.N., Graham S.M. (2006). Memantine MEM-MD-02 Study Group. Behavioral effects of memantine in Alzheimer disease patients receiving donepezil treatment. Neurology.

[5] Leskovjan A.C., Lanzirotti A., Miller L.M. (2009). Amyloid plaques in PSAPP mice bind less metal than plaques in human Alzheimer’s disease. Neuroimage.

[6] Roberts B.R., Ryan T.M., Bush A.I., Masters C.L., Duce J.A. (2012). The role of metallobiology and amyloid-β peptides in Alzheimer’s disease. J. Neurochem..

[7] Good P.F., Perl D.P., Bierer L.M., Schmeidler J. (1992). Selective accumulation of aluminum and iron in the neurofibrillary tangles of Alzheimer’s disease: A laser microprobe (LAMMA) study. Ann. Neurol..

[8] Atwood C.S., Scarpa R.C., Huang X., Moir R.D., Jones W.D., Fairlie D.P., Tanzi R.E., Bush A.I. (2000). Characterization of copper interactions with alzheimer amyloid beta peptides: Identification of an attomolar-affinity copper binding site on amyloid beta1-42. J. Neurochem..

[9] Lang M., Wang L., Fan Q., Xiao G., Wang X., Zhong Y., Zhou B. (2012). Genetic inhibition of solute-linked carrier 39 family transporter 1 ameliorates aβ pathology in a Drosophila model of Alzheimer’s disease. PLoS Genet..

[10] Lovell M.A., Robertson J.D., Teesdale W.J., Campbell J.L., Markesbery W.R. (1998). Copper, iron and zinc in Alzheimer’s disease senile plaques. J. Neurol. Sci..

[11] Crouch P.J., Hung L.W., Adlard P.A., Cortes M., Lal V., Filiz G., Perez K.A., Nurjono M., Caragounis A., Du T. (2009). Increasing Cu Bioavailability inhibits Abeta oligomers and tau phosphorylation. Proc. Natl. Acad. Sci. USA.

[12] Egaña J.T., Zambrano C., Nuñez M.T., Gonzalez-Billault C., Maccioni R.B. (2003). Iron-induced oxidative stress modify tau phosphorylation patterns in hippocampal cell cultures. Biometals.

[13] Kim I., Park E.J., Seo J., Ko S.J., Lee J., Kim C.H. (2011). Zinc stimulates tau S214 phosphorylation by the activation of Raf/mitogen-activated protein kinase-kinase/extracellular signal-regulated kinase pathway. Neuroreport.

[14] Kickstein E., Krauss S., Thornhill P., Rutschow D., Zeller R., Sharkey J., Williamson R., Fuchs M., Köhler A., Glossmann H. (2010). Biguanide metformin acts on tau phosphorylation via mTOR/protein phosphatase 2A (PP2A) signaling. Proc. Natl. Acad. Sci. USA.

[15] Sun X.Y., Wei Y.P., Xiong Y., Wang X.C., Xie A.J., Wang X.L., Yang Y., Wang Q., Lu Y.M., Liu R. (2012). Synaptic released zinc promotes tau hyperphosphorylation by inhibition of protein phosphatase 2A (PP2A). J. Biol. Chem..

[16] Xiong Y., Jing X.P., Zhou X.W., Wang X.L., Yang Y., Sun X.Y., Qiu M., Cao F.Y., Lu Y.M., Liu R. (2013). Zinc induces protein phosphatase 2A inactivation and tau hyperphosphorylation through Src dependent PP2A (tyrosine 307) phosphorylation. Neurobiol. Aging.

[17] Mo Z.Y., Zhu Y.Z., Zhu H.L., Fan J.B., Chen J., Liang Y. (2009). Low micromolar zinc accelerates the fibrillization of human tau via bridging of Cys-291 and Cys-322. J. Biol. Chem..

[18] Huang Y., Wu Z., Cao Y., Lang M., Lu B., Zhou B. (2014). Zinc binding directly regulates tau toxicity independent of tau hyperphosphorylation. Cell Rep..

[19] Soragni A., Zambelli B., Mukrasch M.D., Biernat J., Jeganathan S., Griesinger C., Ciurli S., Mandelkow E., Zweckstetter M. (2008). Structural characterization of binding of Cu (II) to tau protein. Biochemistry.

[20] Walton J.R. (2010). Evidence for participation of aluminum in neurofibrillary tangle formation and growth in Alzheimer’s disease. J. Alzheimers Dis..

[21] Padmanabhan G., Becue I., Smith J.B. (1990). Clioquinol. Anal. Profiles Drug Subst..

[22] Bandyopadhyay S., Huang X., Lahiri D.K., Rogers J.T. (2010). Novel drug targets based on metallobiology of Alzheimer’s disease. Expert Opin. Ther. Targets.

[23] Cherny R.A., Atwood C.S., Xilinas M.E., Gray D.N., Jones W.D., McLean C.A., Barnham K.J., Volitakis I., Fraser F.W., Kim Y. (2001). Treatment with a copper-zinc chelator markedly and rapidly inhibits beta-amyloid accumulation in Alzheimer’s disease transgenic mice. Neuron.

[24] Treiber C., Simons A., Strauss M., Hafner M., Cappai R., Bayer T.A., Multhaup G. (2004). Clioquinol mediates copper uptake and counteracts copper efflux activities of the amyloid precursor protein of Alzheimer’s disease. J. Biol. Chem..

[25] Raman B., Ban T., Yamaguchi K., Sakai M., Kawai T., Naiki H., Goto Y. (2005). Metal ion-dependent effects of clioquinol on the fibril growth of an amyloid {beta} peptide. J. Biol. Chem..

[26] Regland B., Lehmann W., Abedini I., Blennow K., Jonsson M., Karlsson I., Sjögren M., Wallin A., Xilinas M., Gottfries C.G. (2001). Treatment of Alzheimer’s disease with clioquinol. Dement. Geriatr. Cogn. Disord..

[27] Ritchie C.W., Bush A.I., Mackinnon A., Macfarlane S., Mastwyk M., MacGregor L., Kiers L., Cherny R., Li Q.X., Tammer A. (2003). Metal-protein attenuation with iodochlorhydroxyquin (clioquinol) targeting Abeta amyloid deposition and toxicity in Alzheimer disease: A pilot phase 2 clinical trial. Arch. Neurol..

[28] Lannfelt L., Blennow K., Zetterberg H., Batsman S., Ames D., Harrison J., Masters C.L., Targum S., Bush A.I., Murdoch R. (2008). PBT2-201-EURO study group. Safety, efficacy, and biomarker findings of PBT2 in targeting Abeta as a modifying therapy for Alzheimer’s disease: A phase IIa, double-blind, randomised, placebo-controlled trial. Lancet Neurol..

[29] Faux N.G., Ritchie C.W., Gunn A., Rembach A., Tsatsanis A., Bedo J., Harrison J., Lannfelt L., Blennow K., Zetterberg H. (2010). PBT2 rapidly improves cognition in Alzheimer’s Disease: Additional phase II analyses. J Alzheimers Dis..

[30] Adlard P.A., Cherny R.A., Finkelstein D.I., Gautier E., Robb E., Cortes M., Volitakis I., Liu X., Smith J.P., Perez K. (2008). Rapid restoration of cognition in Alzheimer’s transgenic mice with 8-hydroxy quinoline analogs is associated with decreased interstitial Abeta. Neuron.

[31] Finkelstein D.I., Billings J.L., Adlard P.A., Ayton S., Sedjahtera A., Masters C.L., Wilkins S., Shackleford D.M., Charman S.A., Bal W. (2017). The novel compound PBT434 prevents iron mediated neurodegeneration and alpha-synuclein toxicity in multiple models of Parkinson’s disease. Acta Neuropathol. Commun..

[32] Hamano T., Gendron T.F., Causevic E., Yen S.H., Lin W.L., Isidoro C., Deture M., Ko L.W. (2008). Autophagic-lysosomal perturbation enhances tau aggregation in transfectants with induced wild-type tau expression. Eur. J. Neurosci..

[33] Hamano T., Gendron T.F., Ko L.W., Yen S.H. (2009). Concentration-dependent effects of proteasomal inhibition on tau processing in a cellular model of tauopathy. Int. J. Clin. Exp. Pathol..

[34] Hamano T., Yen S.H., Gendron T., Ko L.W., Kuriyama M. (2012). Pitavastatin decreases tau levels via the inactivation of rho/rock. Neurobiol. Aging.

[35] Hamano T., Shirafuji N., Makino C., Yen S.H., Kanaan N.M., Ueno A., Suzuki J., Ikawa M., Matsunaga A., Yamamura O. (2016). Pioglitazone Prevents Tau Oligomerization. Biochem. Biophys. Res. Commun..

[36] Hamano T., Shirafuji N., Yen S.H., Yoshida H., Kanaan N., Hayashi K., Ikawa M., Yamamura O., Fujita Y., Kuriyama M. (2020). Rho-kinase ROCK inhibitors reduce oligomeric tau protein. Neurobiol. Aging.

[37] Shirafuji N., Hamano T., Yen S.H., Kanaan N.M., Yoshida H., Hayashi K., Ikawa M., Yamamura O., Kuriyama M., Nakamoto Y. (2018). Homocysteine Increases Tau Phosphorylation, Truncation and Oligomerization. Int. J. Mol. Sci..

[38] Li W., Jiang M., Xiao Y., Zhang X., Cui S., Huang G. (2015). Folic acid inhibits tau phosphorylation through regulation of PP2A methylation in SH-SY5Y cells. J. Nutr. Health Aging.

[39] Sontag E., Nunbhakdi-Craig V., Lee G., Bloom G.S., Mumby M.C. (1996). Regulation of the phosphorylation state and microtubule-binding activity of Tau by protein phosphatase 2A. Neuron.

[40] Odai T., Terauchi M., Suzuki R., Kato K., Hirose A., Miyasaka N. (2020). Severity of subjective forgetfulness is associated with high dietary intake of copper in Japanese senior women: A cross-sectional study. Food Sci. Nutr..

[41] Gamblin T.C., Chen F., Zambrano A., Abraha A., Lagalwar S., Guillozet A.L., Lu M., Fu Y., Garcia-Sierra F., LaPointe N. (2003). Caspase cleavage of tau: Linking amyloid and neurofibrillary tangles in Alzheimer’s disease. Proc. Natl. Acad. Sci. USA.

[42] Guillozet-Bongaarts A.L., Garcia-Sierra F., Reynolds M.R., Horowitz P.M., Fu Y., Wang T., Cahill M.E., Bigio E.H., Berry R.W., Binder L.I. (2005). Tau truncation during neurofibrillary tangle evolution in Alzheimer’s disease. Neurobiol. Aging.

[43] Kolarova M., García-Sierra F., Bartos A., Ricny J., Ripova D. (2012). Structure and pathology of tau protein in Alzheimer disease. Int. J. Alzheimers Dis..

[44] Ferrer I. (2004). Stress kinases involved in tau phosphorylation in Alzheimer’s disease, tauopathies and APP transgenic mice. Neurotox. Res..

[45] Goedert M., Hasegawa M., Jakes R., Lawler S., Cuenda A., Cohen P. (1997). Phosphorylation of microtubule-associated protein tau by stress-activated protein kinases. FEBS Lett..

[46] Tyagi E., Fiorelli T., Norden M., Padmanabhan J. (2013). Alpha 1-Antichymotrypsin, and Inflammatory Protein Overexpressed in the Brains of Patients with Alzheimer’s Disease, Induces Tau Hyperphosphorylation through c-Jun N-Terminal Kinase Activation. Int. J. Alzheimers Dis..

[47] Yoshida H., Hastie C.J., McLauchlan H., Cohen P., Goedert M. (2004). Phosphorylation of microtubule-associated protein tau by isoforms of c-Jun N-terminal kinase (JNK). J. Neurochem..

[48] Sahara N., Murayama M., Lee B., Park J.M., Lagalwar S., Binder L.I., Takashima A. (2008). Active c-jun N-terminal kinase induces caspase cleavage of tau and additional phosphorylation by GSK-3beta is required for tau aggregation. Eur. J. Neurosci..

[49] Wittmann C.W., Wszolek M.F., Shulman J.M., Salvaterra P.M., Lewis J., Hutton M., Feany M.B. (2001). Tauopathy in Drosophila: Neurodegeneration without neurofibrillary tangles. Science.

[50] Lasagna-Reeves C.A., Castillo-Carranza D.L., Sengupta U., Sarmiento J., Troncoso J., Jackson G.R., Kayed R. (2012). Identification of oligomers at early stages of tau aggregation in Alzheimer’s disease. FASEB J..

[51] Ren Y., Sahara N. (2013). Characteristics of tau oligomers. Front. Neurol..

[52] Patterson K.R., Remmers C., Fu Y., Brooker S., Kanaan N.M., Vana L., Ward S., Reyes J.F., Philibert K., Glucksman M.J. (2011). Characterization of prefibrillar Tau oligomers in vitro and in Alzheimer disease. J. Biol. Chem..

[53] Ward S.M., Himmelstein D.S., Ren Y., Fu Y., Yu X.W., Roberts K., Binder L.I., Sahara N. (2014). TOC1: A valuable tool in assessing disease progression in the rTg4510 mouse model of tauopathy. Neurobiol. Dis..

[54] Konagaya M., Matsumoto A., Takase S., Mizutani T., Sobue G., Konishi T., Hayabara T., Iwashita H., Ujihira T., Miyata K. (2004). Clinical analysis of longstanding subacute myelo-optico-neuropathy: Sequelae of clioquinol at 32 years after its ban. J. Neurol. Sci..

[55] Yassin M.S., Ekblom J., Xilinas M., Gottfries C.G., Oreland L. (2000). Changes in uptake of vitamin b (12) and trace metals in brains of mice treated with clioquinol. J. Neurol. Sci..

[56] Ko L.W., Rush T., Sahara N., Kersh J.S., Easson C., Deture M., Lin W.L., Connor Y.D., Yen S.H. (2004). Assembly of filamentous tau aggregates in human neuronal cells. J. Alzheimers Dis..

[57] Han P., Dou F., Li F., Zhang X., Zhang Y.W., Zheng H., Lipton S.A., Xu H., Liao F.F. (2005). Suppression of cyclin-dependent kinase 5 activation by amyloid precursor protein: A novel excitoprotective mechanism involving modulation of tau phosphorylation. J. Neurosci..

[58] Sahara N., Lewis J., DeTure M., McGowan E., Dickson D.W., Hutton M., Yen S.H. (2002). Assembly of tau in transgenic animals expressing P301L tau: Alteration of phosphorylation and solubility. J. Neurochem..

[59] Bunpo P., Dudley A., Cundiff J.K., Cavener D.R., Wek R.C., Anthony T.G. (2009). GCN2 protein kinase is required to activate amino acid deprivation responses in mice treated with the anti-cancer agent L-asparaginase. J. Biol. Chem..

[60] Wood D.W., Nye J.S., Lamb N.J.C., Fernanez A., Kitzmann M. (2005). Intracellular retention of caveolin 1 in presenilin-deficient cells. J. Biol. Chem..

